# Human Enterovirus B Is a Significant Cause of Aseptic Meningitis and Sepsis-Like Illness in Young Infants in Thailand

**DOI:** 10.7759/cureus.54997

**Published:** 2024-02-26

**Authors:** Jira Chansaenroj, Preeyaporn Vichaiwattana, Jiratchaya Puenpa, Thaksaporn Thatsanathorn, Natthinee Sudhinaraset, Nasamon Wanlapakorn, Yong Poovorawan

**Affiliations:** 1 Center of Excellence in Clinical Virology, Faculty of Medicine, Chulalongkorn University, Bangkok, THA

**Keywords:** echovirus, human enterovirus, infants, sepsis-like illness, aseptic meningitis

## Abstract

Human enterovirus (EV) and Parechovirus (PeV) infections are major causes of sepsis-like illness in infants < 90 days of age. Enterovirus species B (EV-B) was found to be the leading cause of aseptic meningitis in young infants. In Thailand, EV and PeV are not part of the routine screening of blood or cerebrospinal fluid (CSF) of children with suspected aseptic meningitis and sepsis-like illness. Consequently, data on EV and PeV epidemiology are limited. This study tested clinical samples from hospitalized young infants with suspected aseptic meningitis or sepsis-like illness between 2013 and 2022 for EV, PeV, and Herpes simplex virus (HSV). Of 95 specimens, 10 were positive for EV, representing 10.5%. These positive samples included eight CSF and two stool samples. No samples were positive for PeV and HSV. Of these positive cases, EV-B was detected in eight, and EV-A was detected in two cases. The species of EV-B detected include echovirus-18 (E18) (n=2), E21 (n=2), E4(n=1), E5 (n=1), E9 (n=1), and E11 (n=1). Our report demonstrates the significant role of EV-B, and less frequently EV-A, in Thai infants with aseptic meningitis and sepsis-like illness.

## Introduction

Human enterovirus (EV) and Parechovirus (PeV) infections are significant causes of sepsis-like illness in infants < 90 days of age [1]. EVs are also the most frequent cause of pediatric aseptic meningitis [2-4]. Although less frequent, meningitis can be caused by other viruses, including Herpes simplex virus (HSV), West Nile virus, Varicella-zoster virus (VZV), and Cytomegalovirus (CMV) [2,5].

Availability of reverse transcription-polymerase chain reaction (RT-PCR) testing of clinical samples improved the detection and typing of EV and PeV and a better understanding of EV and PeV epidemiology. Several outbreaks of EV-B-associated aseptic meningitis were reported, including the echovirus 30 (E30) outbreak in Bulgaria in 2012 [6] and the E18 nosocomial outbreak in China in 2019 [7]. Severe or potentially fatal neonatal infection was reported to be caused by coxsackievirus B1 (CV-B1) and E11 [8,9]. Other common EV genotypes that cause CNS infection in children are CV-B5, E16, and E11 [10]. Detection of enterovirus by RT-PCR from cerebrospinal fluid (CSF) specimen is recommended for diagnosis of meningitis caused by enterovirus. However, a recent study showed that the detection of EV was more frequent in blood samples than in CSF specimens of infants under two years with fever without source or with sepsis-like illness [11]. Pharyngeal swabs and stools can also be collected and sent for laboratory testing to diagnose EV infection [10].

PeV are RNA viruses that also belong to the Picornaviridae family as EVs. Formerly described as echovirus 22 and 23 in the Enterovirus genus, PeV were reclassified into their Parechovirus genus based on genetic differences and biological properties [12]. PeV infections are prevalent in young children and have been associated with severe diseases such as meningitis, encephalitis, and sepsis-like illness in young infants [13,14]. Our previous studies reported PeV detection in the respiratory and gastrointestinal tract of Thai children presenting with influenza-like illness and acute diarrhea [15].

In Thailand, the screening of blood or CSF in children with suspected aseptic meningitis and sepsis-like illness does not include EV and PeV. As a result, epidemiological data on EV and PeV in young infants in the country are limited. The main aim of this study was to investigate the genotypes of EV and PeV contributing to aseptic meningitis and sepsis-like illness in children under one year of age throughout 2013-2022 and explore possible genetic relatedness between species detected in Thailand and other regions using molecular analysis. These findings may prompt clinicians to pay closer attention to suspected cases of aseptic meningitis and sepsis-like illness through the utilization of molecular techniques.

## Materials and methods

CSF or stool samples were collected from infants with suspected aseptic meningitis or sepsis-like illness and sent for diagnosis at the Center of Excellence in Clinical Virology, Department of Pediatrics, Faculty of Medicine, Chulalongkorn University in Bangkok, Thailand, between January 2013 and December 2022. Clinical samples were screened for EV and PeV by RT-PCR and HSV by PCR. Clinical data of positive cases were retrospectively retrieved. This study was approved by the Institutional Review Board (IRB 002/60) of the Faculty of Medicine at Chulalongkorn University, Bangkok, and authorization for the use of stored samples was granted by the Director of King Chulalongkorn Memorial Hospital. The IRB waived written informed consent because of a retrospective study.

EV and PeV RT-PCRs and HSV PCR were performed in the laboratory of the Center of Excellence in Clinical Virology, Chulalongkorn University. Viral nucleic acid was extracted using the Ribospin vRD kit (GeneAll, Seoul, Korea), following the manufacturer's guidelines. For each case, cDNA was synthesized using the Improm-IITM Reverse Transcription system (Promega, Madison, WI). The hypervariable capsid encodes the VP1 region using a consensus degenerate hybrid oligonucleotide primer (CODEHOP) developed by Nix et al. [16] for detecting EVs. These primers have a broad target specificity for pan-enterovirus that allows for sequencing a segment of VP1 for virtually all EVs found in clinical specimens. For the detection of PeV, the random hexamer primers were used for cDNA synthesis. The nested RT-PCR sets amplify the highly conserved 5’UTR of PeVs that were developed by Harvala et al. [17]. The nested PCR sets to amplify the DNA polymerase gene of HSV were designed by VanDevanter et al. [18]. The PCR products were visualized under UV light after conducting 2% agarose gel electrophoresis. Positive amplicons were directly sequenced and subjected to Basic Local Alignment Search Tool (BLAST) analysis to identify the virus genotype from the sequencing results.

## Results

**Figure 1:**
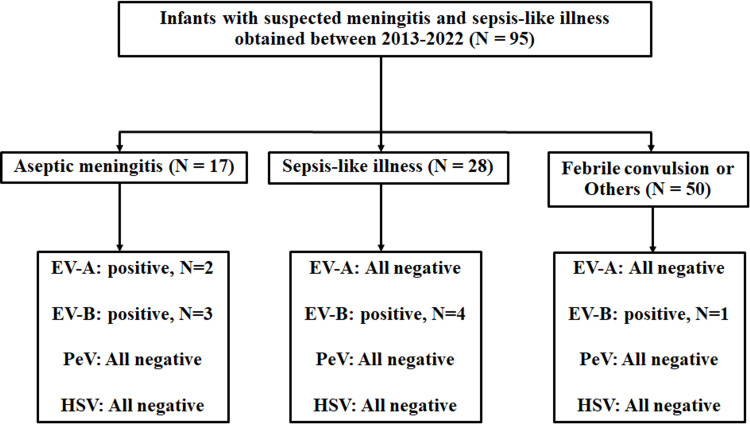
Flowchart for identification of suspected meningitis and sepsis-like illness cases among infants between 2013 and 2022. EV-A = Enterovirus genogroup A, EV-B = Enterovirus genogroup B, PeV = Parechovirus, HSV = Herpes simplex virus

According to Figure 1, 95 specimens of cases with suspected meningitis and sepsis-like illness were screened for EV, PeV, and HSV, and 10 were positive for EV. No sample was positive for PeV and HSV. The sequences of the positive EV detected in this study were deposited and assigned accession numbers OR004179-OR004194 in the GenBank database.

Table 1 displays the clinical characteristics of each positive case, including sex, age, presenting symptoms, final diagnosis, and duration of hospitalization. The age range of the positive cases was from 12 days to one year old. There were five patients diagnosed with aseptic meningitis, four with sepsis-like illness, and one with herpangina. CSF samples were obtained from most patients except for two cases: a patient with herpangina who presented with high-grade fever and seizure and a patient with a sepsis-like illness whose lumbar puncture was unsuccessful. All patients with EV-associated sepsis-like illness (n=4) were infants aged younger than one month. The duration of hospitalization ranged from four to 14 days.

**Table 1:** The clinical characteristics of each positive case. No fatality was found. D = Days, Mo = Months, Y = Years, M = Male, F = Female, CSF = Cerebrospinal fluid

No.	Age at admission	Sex	Admission date	Duration of hospitalization (days)	Date of specimen collection	Final diagnosis	Underlying diseases	Sample type	EV genotype	Accession number
1	13 D	F	21-Sep-15	14	23-Sep-2015	Aseptic meningitis	None	CSF	echo18	OR004179
2	1 Y	M	30-Jan-16	9	1-Feb-2016	Aseptic meningitis	Epilepsy	CSF	CA6	OR004182
3	5 Mo	M	31-Mar-16	9	1-Apr-2016	Aseptic meningitis	None	CSF	CA16	OR004183
4	20 D	M	05-Jul-16	7	6-Jul-2016	Sepsis-like illness	None	CSF	echo18	OR004184
5	18 D	F	12-Jan-17	6	16-Jan-2017	Aseptic meningitis	None	CSF	echo5	OR004187
6	2 Mo	M	07-Mar-17	4	8-Mar-2017	Aseptic meningitis	None	CSF	echo9	OR004188
7	1 Mo	M	04-May-17	5	9-May-2017	Sepsis-like illness	None	CSF	echo4	OR004189
8	9 M	M	22-May-18	5	24-May-2018	Herpangina	None	Stool	echo11	OR004190
9	12 D	M	06-Jul-18	6	9-Jul-2018	Sepsis-like illness	None	CSF	echo21	OR004192
10	1 Mo	F	29-Aug-18	8	3-Sep-2018	Sepsis-like illness	Preterm, tricuspid regurgitation	Stool	echo21	OR004193

**Figure 2:**
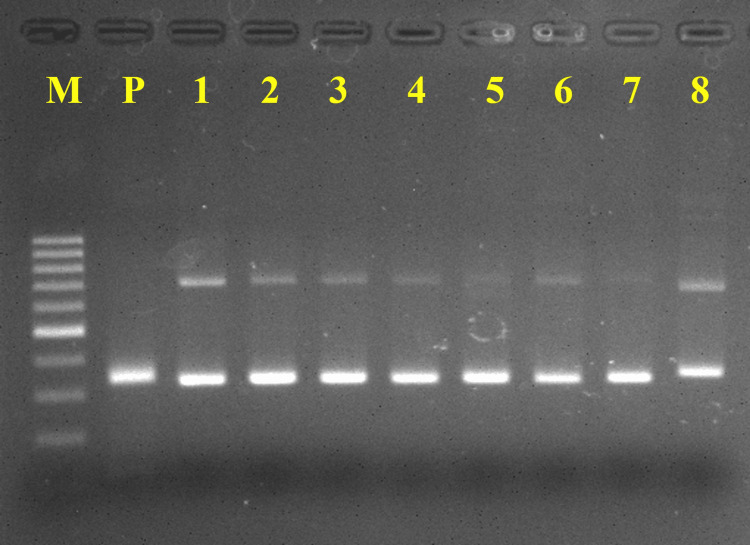
The enterovirus isolated in this study was subjected to electrophoresis on a 2% agarose gel. The amplicon in the VP1 region was obtained using a consensus degenerate hybrid oligonucleotide primer (CODEHOP) developed by Nix et al. [16]. Lane 1, M = 100 base pair marker; Lane 2, P = Positive control; Lane 3, 9 = Positive specimens for enterovirus in this study

According to Figure 2, of the EV-positive cases, EV-B was detected in eight, and EV-A was detected in two cases. The species of EV-B detected include echovirus-18 (E18) (n=2), E21 (n=2), E4 (n=1), E5 (n=1), E9 (n=1), and E11 (n=1). No fatal cases were reported in this study. Each patient successfully recovered and was discharged home.

## Discussion

Our study highlights the circulation of EV-B, and less frequently EV-A, in pediatric cases with aseptic meningitis and sepsis-like illness, as previously noted in other studies [19,20]. The viral strains in this study were very similar to those identified in previous years in China, Taiwan, Vietnam, and Cambodia [21-25]. These have been probably found predominantly in Europe and the Asia-Pacific region [26].

The limitation of this study was that it was a retrospective analysis of convenient samples obtained from one tertiary care center in Bangkok. More extensive studies over more extended periods are needed to understand better EV- and PeV-associated aseptic meningitis and sepsis-like illness epidemiology in Thailand.

## Conclusions

In conclusion, this study highlights that EV-B is the causative agent identified in infant patients with aseptic meningitis and sepsis-like illness in Bangkok, Thailand. These strains were involved with aseptic meningitis in Thailand and continued co-circulations in the region.
